# Microbiological and Toxicological Investigations on Bivalve Molluscs Farmed in Sicily

**DOI:** 10.3390/foods13040552

**Published:** 2024-02-11

**Authors:** Annamaria Castello, Vincenzina Alio, Gaetano Cammilleri, Sonia Sciortino, Andrea Macaluso, Vincenzo Ferrantelli, Sonia Dall’Ara, Fiorella Pino, Irene Servadei, Giuseppa Oliveri, Antonella Costa

**Affiliations:** 1 Istituto Zooprofilattico Sperimentale della Sicilia “A. Mirri”, Via G. Marinuzzi 3, 90129 Palermo, Italyantonella.costa@izssicilia.it (A.C.); 2 Fondazione Centro Ricerche Marine, National Reference Laboratory on Marine Biotoxins, V.le A. Vespucci 2, 47042 Cesenatico, Italyfiorella.pino@centroricerchemarine.it (F.P.); irene.servadei@centroricerchemarine.it (I.S.)

**Keywords:** food safety, shellfish, enteropathogenic *Vibrio*, *Arcobacter* spp., *Aeromonas* spp., *Salmonella* spp., *Escherichia coli*, algal biotoxins

## Abstract

Bivalves can concentrate biological and chemical pollutants, causing foodborne outbreaks whose occurrence is increasing, due to climatic and anthropic factors that are difficult to reverse, hence the need for improved surveillance. This study aimed to evaluate the hygienic qualities of bivalves sampled along the production and distribution chain in Sicily and collect useful data for consumer safety. Bacteriological and molecular analyses were performed on 254 samples of bivalves for the detection of enteropathogenic *Vibrio*, *Arcobacter* spp., *Aeromonas* spp., *Salmonella* spp., and beta-glucuronidase-positive *Escherichia coli*. A total of 96 out of 254 samples, collected in the production areas, were processed for algal biotoxins and heavy metals detection. Bacterial and algal contaminations were also assessed for 21 samples of water from aquaculture implants. *Vibrio* spp., *Arcobacter* spp., *Aeromonas hydrophila*, *Salmonella* spp., and *Escherichia coli* were detected in 106/254, 79/254, 12/254, 16/254, and 95/254 molluscs, respectively. A total of 10/96 bivalves tested positive for algal biotoxins, and metals were under the legal limit. *V. alginolyticus*, *A. butzleri,* and *E. coli* were detected in 5, 3, and 3 water samples, respectively. *Alexandrium minutum*, *Dinophysis acuminata*, *Lingulodinium polyedra,* and *Pseudonitzschia* spp. were detected in water samples collected with the biotoxin-containing molluscs. Traces of yessotoxins were detected in molluscs from water samples containing the corresponding producing algae. Despite the strict regulation by the European Commission over shellfish supply chain monitoring, our analyses highlighted the need for efficiency improvement.

## 1. Introduction

Since the start of aquaculture production’s expansion in the 1990s, bivalve molluscs have been increasingly demanded by consumers for their nutritional value [1]. However, bivalve molluscs can concentrate viruses, bacteria, potentially harmful environmental pollutants, and large quantities of toxic microalgae, the presence of which on the coastlines is one of the greatest risk factors for human health [2,3,4,5,6,7,8,9,10].

The aforementioned issues pose a significant threat to human health, apart from the direct impact on bivalves, that can undergo severe mortalities associated with a variety of pathogens from different taxonomic clades [11]. The scientific literature reports that foodborne pathogens such as *Salmonella* [12] and pathogenic *Vibrio* [13,14] can benefit from warmer temperatures, so more frequent outbreaks can be expected due to climate change that is affecting our planet [14]. *Vibrio* spp. include enteropathogenic species such as *V. vulnificus*, *V. parahaemolyticus*, and *V. cholerae*, but also *V. alginolyticus* and *Vibrio* non O1-non O139, all of which are common causes of disease in humans and aquatic animals worldwide [4,15]. In coordination with the rise in sea surface temperature (SST), additional factors such as the pollution of estuaries and coastal waters from urban or agricultural waste and coastal exploitation may accelerate the increase in pathogen contamination and cause infections in most bivalve species [16]. Also, the scientific literature suggests that climatic changes may influence chemical speciation and the bioaccumulation of trace metals among aquatic organisms [17]. In addition, the temperature-related increase in the uptake and bioaccumulation of metals by bivalves was proven under experimental conditions [18,19]. This evidence stresses the need for a timely and widespread monitoring of metals and pollutants’ accumulation in bivalves.

Global warming also favors the “Harmful Algal Bloom” (HAB), that is, the simultaneous occurrence of marine algal blooms and the proliferation of toxic or harmful species. Although this phenomenon has been known for several years [20], it has been increasingly reported in recent decades and recent studies have highlighted the exacerbating effect of climate change and the warming of marine waters [21,22] on both the variety of harmful algal species and the rate of bloom episodes. In a recent editorial on HABs recorded around the world from 1985 to 2018 [23], Hallegraeff described these events as subjected to discontinuous and heterogeneous fluctuations rather than globally increasing trends, highlighting that different cases of trends and range expansion often remain unexplained. For these reasons, he stressed the urgent need for ecological studies on individual phenomena and improved monitoring and control systems at the local scale.

The aim of this study was to assess the hygienic quality of bivalves farmed for human consumption in Sicily and the occurrence of HAB events along selected Sicilian coastlines in order to collect useful data to ensure consumer safety.

## 2. Materials and Methods

### 2.1. Sampling

A total of 254 samples including mussels (*Mytilus galloprovincialis*, *n* = 229) and clams (*Tapes decussatus*, *n* = 25) were collected in Sicily between November 2017 and April 2021 in shellfish farms and retail outlets by veterinary services as part of the official microbiological control plan implemented in Sicily (Table 1). Molluscs were subjected to both culture and molecular methods for the detection of *Salmonella* spp., enteropathogenic *Vibrio*, *Aeromonas* spp., and *Arcobacter* spp. and the enumeration of beta-glucuronidase-positive *Escherichia coli*.

Molluscs intended for biotoxins and heavy metals detection (92 mussels and 4 clams) were collected from one class C harvesting area (Ganzirri Lake, Messina), from two class B harvesting areas (Siracusa bay and Torre Faro Lake, Messina), and from purification/dispatch centers in Messina and Palermo (Table 2). 

These samples were subjected to the mouse test for the detection of algal biotoxins (PSP, Paralytic Shellfish Poisoning) and to chemical analyses for the detection of lipophilic algal toxins (LTs), water-soluble algal toxins (ASP, Amnesic Shellfish Poisoning), and heavy metals. According to EC Reg 853/2004 [24], the LTs include okadaic acid (OA) dinophysistoxins (DTXs), yessotoxins (YTXs), and azaspiracids (AZAs). Moreover, a subsample of 21 water samples, collected from aquaculture implants along the east coast of Sicily, were subjected to bacteriological examinations and to microscopy analysis for the detection of biotoxin-producing algae. All samples were taken under sterile conditions, kept refrigerated (T = 4 °C), and processed within the following 24 h.

### 2.2. Microbiological Analyses

#### 2.2.1. Bacteriological Examinations

Referring to molluscs, the content of each bivalve (flesh and intravalvular liquid) was transferred into stomacher bags and then mashed in a stomacher for 2 min. Water samples were filtered using one 0.45 µm nitrocellulose membrane filter (Sartorius, Göttingen, Germany) per 200 mL of sample. The homogenate and filters were processed as described below.

##### Detection of *Salmonella* spp.

*Salmonella* spp. were detected using the conventional culture method, immunoenzymatic assay, and biomolecular assay. Regarding the water samples, only the culture method was used.

- Conventional culture method

According to ISO 6579-1:2017 [25], sample processing was performed as follows: 25 g of homogenate of molluscs was diluted 1/10 (*w*/*v*) in buffered peptone water (BPW) and incubated at 37 °C for 24 h. Filters of water samples were instead placed into sterile bags containing 30 mL of BPW and incubated at 37 °C for 24 h (pre-enrichment). Two steps of selective enrichment were performed as follows: 0.1 mL of the pre-enriched inoculum was transferred to 10 mL of Rappaport–Vassiliadis Soy broth (RVS) and incubated at 41.5 °C for 24 h, and 1 mL of the pre-enriched inoculum was transferred to 10 mL of Muller–Kauffman tetrathionate novobiocin broth (MKttn) (Oxoid, UK) and incubated at 37 °C for 24 h. After enrichment, a loopful of each inoculum was streaked on two selective media (XLD: Xylose Lysine Deoxycholate agar and BGA: Brilliant Green Agar (Oxoid, UK)) and incubated at 37 °C for 24 h. Presumptive colonies were picked from positive *Salmonella* cultures and screened further for biochemical characterization. The latter was obtained using conventional methods (triple sugar iron-TSI, urease agar, lysine decarboxylation, oxidase test) or using the API 20E biochemical identification strips (bioMérieux, Marcy L’etoile, France) and serotyping, according to the Kauffmann–White–Le Minor scheme.

- ELFA Immunoenzymatic assay

Sample processing was performed as follows: 25 g of homogenate was diluted 1/10 (*w*/*v*) in selective enrichment broth and incubated at 41.5 °C for 24 h. An amount of 0.5 mL of the suspension was processed using the VIDAS Salmonella UP kit (AFNOR BIO-12/32-10/11, ISO 16140) [26] and the automatic immunoassay system MiniVIDAS (bioMérieux, Marcy L’etoile, France) as recommended by the manufacturer. Positive samples were isolated on selective media and processed further for biochemical characterization and serological confirmation, as mentioned above.

- Real-Time PCR

According to the Official Method AOAC 120301:2003, sample processing was performed according to Lindhardt et al. 2009 [27] as follows: DNA was extracted from the pre-enriched inoculum (see conventional culture method) using the Foodproof ShortPrep I kit (Biotecon Diagnostic, Potsdam, Germany), according to the manufacturer’s instructions. The target DNA was detected using the thermal cycler LightCycler 2.0 (Roche Applied Science, Penzberg, Germany) and the Foodproof Salmonella Detection kit Hybridization Probes (Biotecon Diagnostic), as recommended by the manufacturer. Positive samples were isolated on selective media and processed further for biochemical characterization and serological confirmation, according to ISO 6579-1:2017 [25].

##### Detection of Enteropathogenic *Vibrio* spp.

- Conventional culture method

According to ISO 21872-1:2017 [28], sample processing was performed as follows: 25 g of homogenate was diluted 1/10 (*w*/*v*) in alkaline saline peptone water (ASPW) and incubated at 37/41.5 °C overnight (pre-enrichment). Selective enrichment was performed as follows: 1 mL of the pre-enriched inoculum was diluted 1/10 (*v*/*v*) in ASPW and incubated at 37/41.5 °C for 18 h. At the end of the incubation period, an inoculating loop of the culture broth was seeded onto thiosulfate citrate bile salt agar (TCBS) (Microbiol S.r.l., Macchiareddu, Italy) and CHROMagar Vibrio (CHROMagar^TM^, Saint-Denis, France) plates. Water sample filters were placed on each agar plate. After incubation at 37 °C for 24 h, presumptive colonies were characterized by the following laboratory protocol, oxidase test, Gram staining, lysine decarboxilase-LDC, arginine dehydrolase-ADC, β-galattosidase, indole test, and salt tolerance, following the API 20NE identification system (BioMerieux, Marcy l’Etoile, France). All isolates presumptively identified as *Vibrio parahaemolyticus*, *V. cholerae,* and *V. vulnificus* were confirmed and further characterized by means of molecular tests.

- Molecular tests

Isolated colonies were picked from agar nutrient plates and resuspended in 1 mL of saline solution. After incubation at 95 ± 2 °C for 5 ± 1 min, samples were centrifuged at 1000× *g* for 1 min. DNA contained in the supernatant was further processed by multiplex PCR for the detection of highly conserved species-specific marker genes [4], namely *tox*R for *V. parahaemolyticus* [29], *prVC* (16S-23S rRNA intergenic spacer region) for *V. cholerae,* and *vvha* for *V. vulnificus*. A separated PCR assay [30] was applied to detect the virulence genes associated with enteropathogenicity of *V. parahaemolyticus* (*tdh* and *trh*). Each batch of PCR assays included DNA extracted from certified strains as positive controls (*V. parahaemolyticus* NTCT 10884-ATCC 17802, *V. vulnificus* ATCC 27562, and *V. cholerae* ATCC 1473A) and molecular-grade water as the negative control. After PCR amplification, 10 μL of each reaction product was loaded into 2.0% agarose gels in Tris-acetate-EDTA buffer (Thermo Fisher, Waltham, MA, USA), containing 5 µL SYBR^®^ Safe DNA gel stain (Thermo Fisher, Waltham, MA, USA). Amplicons were visualized under a UV transilluminator (GelDoc-It Imaging System, UVP LLC, Upland, USA).

##### Detection of *Aeromonas* spp.

*Aeromonas* spp. were detected using a self-developed method [31], appropriately modified. This included the following steps: selective enrichment in alkaline saline peptone water (ASPW), inoculation of the selective media Aeromonas agar (Microbiol S.r.l.) and thiosulfate citrate bile salt agar (TCBS) with the enriched broth, and incubation at 28 °C for 24–48 h. Suspected colonies were inoculated onto trypticase soy agar (TSA) and further characterized by the following laboratory protocol, catalase and oxidase test, Gram staining, and salt tolerance, by the API 20NE identification system (BioMerieux, Marcy l’Etoile, France). 

##### Detection of *Arcobacter* spp.

- Conventional culture method

The homogenate (25 g) was diluted 1/10 (*w*/*v*) in Arcobacter enrichment broth added with cefoperazone, amphotericin B, and teicoplanin (CAT-Oxoid, Basingstoke, UK) and incubated at 30 °C for 48 h under microaerophilic conditions [32]. Filters of water samples were placed into sterile bags containing 30 mL of Arcobacter enrichment broth added with CAT and incubated at 30 °C for 48 h under aerobic conditions. At the end of the incubation period, 200 µL of the enriched broth was dropped on the surface of a 0.45 µm nitrocellulose membrane filter (Sartorius, Göttingen, Germany), placed onto two selective agar plates: trypticase soy agar (TSA) plus 5% laked horse blood (Oxoid, Basingstoke, UK) + CAT and modified charcoal cefoperazone deoxycholate (mCCDA) + CAT. Plates were incubated at 30 °C for 1 h and, after removal of the filters, at 30 °C for 48 h under aerobic conditions [33]. Presumptive colonies grown within the filter area were subcultured onto blood agar plates and further characterized by the following laboratory protocol: catalase and oxidase test, Gram staining, motility test, salt tolerance, and urease test. Isolates referred to *Arcobacter* (oxidase- and catalase-positive, Gram-negative, spiral-shaped, motile, urease-negative) were further processed using multiplex PCR for species identification.

- Multiplex PCR

DNA extraction was performed following the protocol developed by Houf et al. [34] and described by Ertas et al. [35]. *A. butzleri*, *A. cryaerophilus,* and *A. skirrowii* were simultaneously identified using a multiplex PCR assay targeting the 16S rRNA and 23S rRNA genes [33]. Each batch of analysis included DNAs extracted from the certified strains *A. butzleri* (NCTC 12481), *A. cryaerophilus* (NCTC 11885), and *A. skirrowii* (NCTC 12713) as positive controls and PCR-grade water as the negative control.

PCR products were separated by electrophoresis on 1.5% agarose gel, stained with an SYBR-safe DNA gel stain. Bands were visualized under the UV transilluminator (GelDoc, Euroclone, Pero, Italy) and their size was compared to the expected ones, obtained from positive controls (401 bp from *A. butzleri*, 257 bp from *A. cryaerophilus*, and 641 bp from *A. skirrowii*).

##### Enumeration of Beta-Glucuronidase-Positive *Escherichia coli*

The bivalve samples were examined using the Most Probable Number (MPN) method in accordance with the EU reference method ISO 16649-3:2015 [36]. Briefly, the homogenate was diluted 1:10 in Peptone Salt Solution (PSS) and 1 + 9 serial dilutions were prepared. Ten milliliters of the 1:10 suspension was transferred to each of three tubes of double-strength Mineral-Modified Glutamate Medium (MMGB) (Oxoid, Basingstoke, UK). Aliquots of 1.0 mL of the 1:10 suspension and the 1 + 9 serial dilutions were transferred to each of the three tubes of single-strength MMGB. All the double- and single-strength MMGB tubes were incubated aerobically at 37 °C for 24 h. Subcultures from positive MMGB tubes that changed color from purple to yellow were plated on chromogenic Tryptone Bile Glucuronide Agar (TBX) plates (Oxoid, Basingstoke, UK) incubated aerobically at 44 °C for 20 h. At the end of incubation, the number of positive tubes of double- or single-strength MMGB tubes were counted in order to estimate the level of *E. coli* (MPN)/100 g according to ISO 7218, 2007 [37].

Water samples were processed using the quantitative method Colilert^®^-18/Quanti-Tray (IDEXX), according to ISO 9308-2: 2012 [38], and the results are reported as *E. coli* (MPN)/100 mL.

#### 2.2.2. Detection of Algal Biotoxins

##### Paralytic Shellfish Toxins (PSTs)

PSTs were detected according to the Official Method AOAC 959.08 [39], under application until October 2021. Namely, the molluscs were cleaned with fresh water and opened by cutting the adductor muscle. The meat was removed and transferred to a sieve, letting drain for 5 min. An amount of 100 g of homogenate was subjected to chemical extraction using 100 mL of HCL 0.1 M. After adjusting the pH to 3.0, the mixture was boiled for 5 min and let cool at room temperature. The pH value was restored to 3.0 by adding HCl 0.5 M or NaOH 0.1 M and the mixture was diluted up to a final volume of 200 mL. After centrifugation at 3000 rpm for 5 min, the supernatant (acid extract) was collected and 1 mL was inoculated in two mice (weighing 19–21 g) by intraperitoneal injection. The remaining extract was stored at −20 °C for further analyses. Symptoms were monitored for 60 min and the time of death was recorded. When mice survived beyond 60 min, the PSP content was reported as “undetectable”. Positive samples induced death within 60 min and the occurrence of specific symptoms such as neurologic and/or respiratory disruption. High concentrations of saxitoxins generally induce death within 5–10 min [40]. An aliquot of positive samples was sent to the National Reference Laboratory (NRL) in Cesenatico for confirmation and quantitation as described before [41]

##### Microscopy Analysis for the Detection of Biotoxin-Producing Algae in Water

The samples (1 L) were taken manually in clean polyethylene bottles at a depth of 1, 7, and 14 m from the water surface and fixed with Lugol’s iodine solution in order to preserve them for further analysis, carried out at NRL on Marine Biotoxins (Cesenatico) according to the EU reference method UNI EN ISO 15204:2006 (Utermöhl’s method, 1958) [42]. The PSTs producing microalgal species were counted using settling chambers (25–50 mL) under an inverted Nikon microscope (Eclipse Ti- U, objectives: 20× CFI planapo, 40× CFI planapo, and 100× CFI planfluor oil) equipped with digital cameras (Nikon DSFi2) and NIS-Elements imaging software (version n. 4.20.00). The morphology of fixed cells was also analyzed with an Ultraviolet 100 W Mercury lamp. Plate patterns were studied according to Balech [43] after staining with Calcofluor white (Fluorescent Brightener 28, Sigma, Steinheim, Germany) and was observed under UV epifluorescence (the UV filter arrangement was for 330–380 nm excitation and 420 nm emission wavelength). Microalgal abundance was expressed as cell numbers per liter (cells/L). The quantitative detection limit for *Alexandrium* species count by Utermöhl’s method was 120 cells/L for 25 mL subsamples or 60 cell/L for 50 mL subsamples, and the level of significance was 0.05 for both.

##### Chemical Analyses

Referring to reagents and standards used for chemical analyses, ultrapure water was obtained from the Milli Q purification system (Merck, Darmstadt, Germany). Acetonitrile, ammonium format, nitric acid, methanol, trifluoroacetic acid, and formic acid were purchased from Sigma-Aldrich (Amsterdam, The Netherlands). Liposoluble toxin standard solution of okadaic acid, dinophysis toxins, azaspiracids, pectenotoxins, and yessotoxins was purchased from LGC (Teddington, UK). Domoic acid standard was purchased from Sigma-Aldrich (Amsterdam, The Netherlands). Cr, Mn, As, Cd, and Pb standards were purchased from Sigma-Aldrich (Amsterdam, The Netherlands). All the reference standards were stored at −20 °C. Below a list of of the chemicala analyses performed.

- Algal toxin analysis

LTs (OA, DTXs, YTXs, AZAs) were assessed using an LC-MS/MS method according to EU Regulation n. 15/2011 [44]. Samples were extracted as described by the European Union Reference Laboratory for Marine Biotoxins [45]. An ACCELA TSQ Vantage with an ACCELA AS autosampler (Thermo Fisher, Waltham, MA, USA) was used for the determination. The separations were carried out using an XBridge C18 column (3.5 μm 4.6 × 150 mm column, Waters, Yorba Linda, CA, USA) The assessment of water-soluble algal toxins (domoic acid-ASP) was performed using an HPLC according to the AESAN-EURLMB 2008, reference method [46]. The separations were carried out with a Prodigy ODS-3 column (5 µm 250 × 4.6 mm, Phenomenex, Torrance, CA, USA) on an Agilent 1200 HPLC with a DAD detector for the quantification. The results of the validation of the LC-MS/MS method are shown in Table 3. The linearity test produced satisfactory results, with r^2^ > 0.997 for all the toxins considered.

- Toxic metals and metalloids analysis

The detection of Cr, Mn, As, Cd, and Pb was performed using an Inductively Coupled Plasma-Mass Spectrometry (ICP-MS) method according to protocols reported before [47,48]. The sample extraction was carried out using a digestion procedure through a Multiwave 3000 digestor (Anton-Paar, Graz, Austria) in accordance with the UNI EN 13805:2002 [49]. The samples digested were made up to a 50 mL volume with Millipore deionized water until the ICP-MS analysis. The analysis was carried out on a 7700× series ICP-MS (Agilent Technologies, Santa Monica CA, USA) with the operating conditions and instrumental settings reported by Cammilleri et al., 2020 [48]. Mercury (Hg) determination was performed using a DMA-80 thermal direct mercury analyzer (Milestone GmbH, Germany) [48] based on atomic absorption spectroscopy at a fixed wavelength of 250 nm. All the analytical procedures were validated according to the ISO 17025:2018 [50] and the Commission Decision 2002/657 for linearity, recovery, limit of detection (LOD), and limit of quantification (LOQ). The validation of the methods produced satisfactory results, showing a linearity r^2^ > 0.996 for all the elements examined and a recovery range between 95% and 113.6%. The LOD and LOQ values for all the elements are reported as follows: 0.005 mg/Kg and 0.02 mg/Kg for As; 0.005 mg/Kg and 0.02 mg/Kg for Cd; 0.001 mg/Kg and 0.002 mg/Kg for Pb; 0.07 mg/Kg and 0.09 mg/Kg for Cr; and 0.07 mg/Kg and 0.09 mg/Kg for Mn.

##### Data Analysis

The prevalence of *Vibrio* spp., *Aeromonas* spp., *Arcobacter* spp., *Salmonella* spp., and *E. coli* was calculated with the 95% confidence interval (CI). The comparison of the prevalence was carried out using the chi-squared test or Fisher exact test when needed. A probability value (*p*-value) of 0.05 was regarded as statistically significant.

## 3. Results

### 3.1. Bacteriological Examinations

*Vibrio* spp. were detected in 106 out of 254 bivalves (41.73%, 95% CI: 35.67% to 47.79%). Positive isolates also occurred in samples collected in winter, hence indicating that seawater temperatures were suitable for their proliferation. Significant differences between mussels and clams were revealed for the prevalence of *Vibrio* spp. and *E. coli.* The majority of isolates tested positive for *V. alginolyticus* (34.06% of mussels and 12% of clams), while *V. parahamolyticus* was detected in 18.8% of mussels and 12% of clams. None of the *V. parahaemolyticus* isolates tested positive for the molecular markers *tdh* and *trh. Aeromonas* spp. were detected only in 12 out of 229 mussels, and the positive strains were identified as *Aeromonas hydrophila* and isolated together with *V. alginolyticus* (Table 4).

*Arcobacter* spp. were detected in 79 out of 254 bivalves (31.1%, 95% CI: 25.41% to 36.79%) and positive isolates included *A. butzleri* (21.39% of mussels and 32% of clams), *A. cryaerophilus* (7.86% of mussels and 8% of clams), and *A. skirrowii* (8% of clams). Nine mussels and two clams tested positive for *Arcobacter butzleri*, *V. parahaemolyticus,* and *V. alginolyticus. Salmonella* spp. were detected in 6.3% of bivalves, sampled mostly at dispatch centers. *E. coli* was detected in 37.4% of bivalves (95% CI: 31.45% to 43.35%) (Table 5).

Referring to seawater collected at aquaculture implants together with bivalves, 5/21 samples tested positive for *V. alginolyticus*, 3/21 tested positive for *A. butzleri,* and 3/21 tested positive for *E. coli*.

### 3.2. Detection of Toxin-Producing Algae, Algal Biotoxins, and Toxic Metals

A total of 10 out of 96 bivalves (14.3%) collected at two sampling points in the class B harvesting area of Syracuse bay tested positive for PSTs (mouse test). In particular, 7/10 positive samples induced the death of mice early after jugular injection (<10 min), and their saxitoxins content, detected using LC MS/MS, was over the legal limit (800 µg STXeq/kg): PST analysis conducted by the NRL of Cesenatico detected a maximum toxicity of 8.139 µg STXeq/kg e.p. [41]. Seawater samples, collected together with the PST-containing mussels, were subjected to microscopy analyses, revealing the saxitoxin-producing algae *Alexandrium minutum* Halim with values between 60 and 5400 cell/L [41].

Other algal species were detected in these water samples, as well as *Dinophysis acuminata* (60–140 cells/L), *Lingulodinium polyedra* (100–3200 cells/L), and *Pseudo-nitzschia* spp. (2000–300,000 cells/L) which produce Diarrhetic Shellfish Poisons (DSPs), YTXs, and ASPs respectively.

No domoic acid was detected, whereas traces of YTXs (4–56 µg/Kg) (<LOD of the method) were detected in water samples containing the corresponding producing algae.

No toxic algal species in the water samples and algal biotoxins in bivalves were detected in harvesting areas of Messina (Ganzirri Lake and Faro Lake).

No toxic metals and metalloids were detected in all the samples examined (<LOD of the method; Table 6).

## 4. Discussion

Over the 20th century, the Earth’s average surface temperature has increased by 0.76 °C and it is likely to further rise by another 1.8 °C (best-case scenario) to 4.0 °C (worst-case scenario) by the end of the 21st century [51]. The effects of seawater warming, together with pollution and coastal exploitation, can expose humans to food hazards and pose a risk for aquatic species. In fact, the aforementioned phenomena can promote the proliferation of potentially pathogenic microorganisms and toxin-producing algae as well as favor the uptake and bioaccumulation of biotic and abiotic pollutants especially by bivalve molluscs.

Climate change, pollution, and coastal exploitation are hardly reversible; therefore, scientists’ efforts should be directed towards the effective monitoring of their impact on aquatic environments, in order to mitigate their detrimental effects against animal and human health.

The positive influence of water warming on the proliferation of pathogenic *Vibrio* and *Salmonella* was reported by various authors [12,13,14,52,53]. *V. cholerae* non O1/O139 and other pathogenic *Vibrio* spp. are spread worldwide, and even though they are rarely or occasionally associated with disease, the occurrence of outbreaks is increasing over time, also because of the progressive rise in sea surface temperature [54]. In spite of their spread, Europe lacks mandatory notification systems for Vibrio-associated illnesses other than those caused by *V. cholerae* O1/O139, and this prevents an accurate estimation of the number of infections. Moreover, current European guidelines for foodstuffs do not include their assessment among the microbiological criteria required for food.

Many authors have reported the isolation of pathogenic *V. parahaemolyticus* from the Adriatic Sea [55] and along the Italian coastlines [56,57,58,59], with variable rates of contamination. In particular, Caburlotto et al. [57] recorded values of about 6–9% in water and mussels, while Ottaviani et al. [59] recorded contamination rates accounting for 61.4% in mussels and 8.8% in clams.

The contamination rates recorded in this study are much lower than those found by Lamon et al. [60] in bivalves collected along the Sardinian coastlines (96% pathogenic *Vibrio* spp. in mussels and 77% in clams); nevertheless, we can confirm that pathogenic *Vibrio* can be isolated from seafood also during colder seasons (late autumn and winter), suggesting that seawater temperatures are sufficiently warm for their proliferation also during these seasons, previously considered less conducive to their proliferation [61]. Contaminated samples were detected in both breeding/housing systems and retail outlets, confirming the ineffectiveness of current depuration systems against *Vibrio* spp., already suggested by others [60,62].

Our analyses revealed low rates of contamination for *Salmonella* spp. (6.3%) in bivalves collected from retail outlets, demonstrating the risk of recontamination while processing or selling.

*Arcobacter* spp. were detected in 31.1% of bivalves, with higher rates in clams (58%) compared to mussels (29.2%). Therefore, our results confirm the spread of these emerging pathogens in seafood and underline the need for further studies and an effective human risk assessment [63]. Most of isolates were identified as *Arcobacter butzleri*, which is classified as a dangerous agent for human health by the International Commission on Microbiological Specifications for Foods [64]. The fecal indicator bacterium (FIB) *Escherichia coli* was detected in 37.4% of bivalves and in 14.3% of water samples collected at aquaculture implants. The maximum number of fecal indicators allowed for bivalve molluscs is regulated by the European Commission (EU Regulation 2015/2285) [65] and the microbiological criteria of aquaculture implants are strictly monitored by official controls. Still, the spread of antibiotic resistance among strains of *E. coli* is widely recognized and a large body of evidence demonstrated that *E. coli* can persist outside the hosts in many environmental reservoirs such as beach sands [66,67], marine sediments, and wetlands [68,69,70]. Moreover, this bacterium is able to produce biofilm on various macroalgae and to constitute heterogeneous bacterial populations [71]. Antibiotic resistance may be transmitted by *E. coli* to any coexisting bacteria from the same biofilm population, even the potentially pathogenic ones. For this reason, the effective monitoring of the aquaculture system should also evaluate the spread of antibiotic resistance among FIBs, apart from their quantification. Climatic changes are also known for having increased the occurrence of algal blooms and favored their spread beyond tropical regions over the last two decades [20,21]. The co-occurrence of *Alexandrium* species and algal toxins (PSTs) along the Italian coastlines has been known for several years, in particular in the Ionian Sea [72,73,74] where recurrent algal blooms were recorded annually and mostly in spring [72]. But more recently, other toxic species such as *A. pacificum* Litaker, 2014 (former *A. catenella* (Whedon & Kof.) Balech, 1985), were identified apart from *A. minutum* Halim and PSP-positive samples, which were detected between late winter and early spring [41,73,75]. Contamination by the PSP toxin in farmed mussels, with values beyond the limits established by law, leads to the consequent immediate closure of the production area.

Our study did not reveal concentrations over the legal limit for any of the heavy metals assessed. Significantly high values of contamination were reported by other authors in other geographic areas [76,77], while scarce data are available referring to the central Mediterranean Sea. A study carried out in Australia demonstrated that the accumulation of metals in water and their consequent bioaccumulation in bivalves can be influenced by various weather factors, such as the dry winds and humidity, which promote the transport into the waterways of water-insoluble and water-soluble metals, respectively [78]. Apart from the aforementioned data, many aspects of this phenomenon are still unclear and the monitoring of its spread should be implemented.

## 5. Conclusions

The results of this study confirmed that bivalve molluscs can be easily contaminated and drive pathogenic bacteria and toxins to humans. Potentially pathogenic bacteria were also detected in bivalves collected at retail outlets, suggesting the need to further implement the monitoring systems along the entire aquaculture supply chain up to retail, without underestimating the role played by water. This is also in light of the climatic changes affecting our planet, which constitute a significant challenge in the field of food safety and is worth the attention of both scientists and legislators.

Although this study did not reveal worrying levels of metals, highly contaminated molluscs were found by others in other areas of the Mediterranean Sea and in the Adriatic Sea. These data suggest the need for a continuous monitoring and further studies, in order to investigate the multiplicity of factors that can influence metals accumulation in aquatic environments.

## Figures and Tables

**Table 1:** Bivalve specimens subjected to bacteriological analyses.

Sample		N	Sampling Sites	
Taxonomic Name	Common Name		Shellfish Farms	Retail Outlets
*M. galloprovincialis*	Mussels	229	92	138
*T. decussatus*	Clams	25	4	20
Total number		254	96	158

N, number of samples.

**Table 2:** Bivalve specimens subjected to biotoxins and heavy metals detection.

Sample		N	Sampling Sites		
Taxonomic Name	Common Name		Class C ^1^	Class B ^2^	Purification/ Dispatch ^3^
*M. galloprovincialis*	Mussels	92		72	20
*T. decussatus*	Clams	4	4		
Total number		96	4	72	20

^1^ Class C harvesting areas. ^2^ Class B harvesting areas. ^3^ Purification/dispatch centers.

**Table 3:** Validation of LC-MS/MS for the detection of the algal toxins analyzed.

Concentration Level (µg/Kg)	Repeatability (µg/Kg)	LOD (µg/Kg)	LOQ (µg/Kg)	Mean Recovery (%)
OA				
40	7.6	21.6	26.4	77
160	14.6			
DTX1				
40	5.1	23.3	28.3	114
160	19.1			
DTX2				
40	18.9	24	27	96
160	12.3			
AZA1				
40	6	9.1	10.6	60
160	16.4			
AZA3				
40	4.3	6.5	8.1	70
160	11.2			
PTX2				
40	7.5	18.4	26.2	63
160	13			
YTX				
100	11.7	55	61.2	78
400	45.3			

**Table 4:** Samples tested positive for *Vibrio* spp. and *Aeromonas* spp.

Sample	N	*Vibrio* spp. (%)		*Aeromonas* spp. (%)	
		P	Species	P	Species
*M. g.* ^1^	229	25 (10.92%)	*V. parahaemolyticus*	12 (5.24%)	*A. hydrophila*
		60 (26.2%)	*V. alginolyticus*		
		18 (7.86%)	*V. parahaemolyticus* *+ V. alginolyticus*		
*T. d.* ^2^	25	3 (12%)	*V. parahaemolyticus* *+ V. alginolyticus*		
Total number	254	106 (41.73%)		12 (4.72%)	

^1^ *M. galloprovincialis*. ^2^ *T. decussatus*. N, number of samples. P, positive samples.

**Table 5:** Samples tested positive for *Arcobacter* spp., *Salmonella* spp., and *E. coli*.

Sample	N	*Arcobacter* spp. (%)		*Salmonella* spp. (%)		*E. coli* (%)
		P	Species	P	Species	P
*M. g.* ^1^	229	49 (21.39%)	*A. butzleri*	4 (1.75%)	*S. typhimurium*	91 (39.73%)
				4 (1.75%)	*S. bredeney*	
				2 (0.87%)	*S. derby*	
		18 (7.86%)	*A. cryaerophilus*	2 (0.87%)	*S. corvallis*	
				2 (0.87%)	*S. kasenyi*	
*T. d.* ^2^	25	8 (32%)	*A. butzleri*	2 (8%)	*S. enterica* subsp. *Enterica serovar Salamae*	4 (16%)
		2 (8%)	*A. cryaerophilus*			
		2 (8%)	*A. skirrowii*			
Total number	254	79 (31.1%)		16 (6.3%)		95 (37.4%)

^1^ *M. galloprovincialis*. ^2^ *T. decussatus*. N, number of samples. P, positive samples.

**Table 6:** Samples tested for PSTs, LTs, ASP, and heavy metals.

Sample	Sampling Sites	N	PSTs (Mouse Test)	LTs	ASP	Heavy Metals
*M. g.* ^1^	Porto Grande, SR (Class B)	70	10 (14.3%)	<LOQ	U	<LOD
*M. g.* ^1^	Torre Faro, ME (Class B)	2	-	<LOQ	U	<LOD
*M. g.* ^1^	Purification/dispatch centers	20	-	<LOQ	U	<LOD
*T. d.* ^2^	Ganzirri, ME Breeding center	4	-	<LOQ	U	<LOD
Total number		96	10 (10.4%)			

^1^ *M. galloprovincialis*. ^2^ *T. decussatus*. N, number of samples. LOQ, limit of quantification. U, undetected. LOD, limit of detection.

## Data Availability

The original contributions presented in the study are included in the article, further inquiries can be directed to the corresponding author.
